# Number of Readmissions and Its Determinants Among Patients With Heart Failure at Referral Hospitals in Amhara Region, Northwest Ethiopia: A Cross‐Sectional Study Using Zero‐Inflated Negative Binomial Model, 2023

**DOI:** 10.1002/hsr2.70408

**Published:** 2025-01-31

**Authors:** Mihretie Gedfew, Bekele Tesfaye, Haile Amha, Tirusew Wondie, Getnet Gedif, Wodajie Gietaneh, Tadesse Yirga Akalu, Lieltework Yismaw, Gedefaw Diress

**Affiliations:** ^1^ College of Health Science Debre Markos University Debre Markos Ethiopia

**Keywords:** Ethiopia, patients with heart failure, readmissions, zero inflation negative binomial regression

## Abstract

**Background:**

Heart failure is a leading cause of hospital readmissions in the Amhara region, Northwest Ethiopia.

**Aim:**

This study aimed to determine the number of readmissions and identify the determinants among patients with heart failure at referral hospitals in the Amhara region, Northwest Ethiopia, in 2023.

**Methods:**

A cross‐sectional study was conducted with 663 heart failure patients in Amhara region referral hospitals from September 2022 to February 2023. Simple random sampling was used for patient selection, and data were collected through chart reviews and interviewer‐administered questionnaires. Zero‐inflated negative binomial models were applied for data analysis. Data collection tools were pre‐tested for reliability and validity.

**Results:**

Among 663 patients, 237 (35.7%) were readmitted at least once. An increased respiratory rate (IRR = 1.015; 95% CI: 1.0004, 1.031; *p* < 0.044) and longer medication duration (IRR = 1.011; 95% CI: 1.016, 1.051; *p* < 0.0001) were associated with more readmissions. Patients with poor social support had 59.4% fewer readmissions compared to those with good social support (IRR = −1.595; 95% CI: −0.02, −0.005; *p* < 0.041). A higher body mass index (IRR = 0.115; 95% CI: 0.035, 0.196; *p* < 0.004) was linked with a higher likelihood of remaining in the “always‐zero” group, while an increased pulse rate reduced the odds (IRR = −0.013; 95% CI: −0.025, −0.008; *p* < 0.036). The mortality rate among readmitted patients was 11.39%.

**Conclusion:**

This study found significant readmission rates among HF patients. Factors such as respiratory rate and medication duration were linked to increased readmissions, while poor social support was associated with fewer readmissions, this likely reflects limited healthcare access in low‐ and middle‐income countries among individuals with lower social support. The high mortality rate underscores the need for targeted interventions to improve patient outcomes.

## Summary

What Is Known on This Topic?

- Heart failure (HF) remains a significant health issue, yet the extent of readmissions among HF patients is not well understood.
- HF is responsible for nearly half of all deaths related to noncommunicable diseases, and over 20% of HF patients are thought to be readmitted within 30 days of discharge.
- Addressing these high readmission rates is critical for improving patient outcomes and reducing the burden on healthcare systems.

What This Study Adds?

- This study aims to determine the readmission rates among patients with HF, which is crucial for developing interventions that reduce hospital readmissions and their impact on patients; and families' socioeconomic well‐being.
- Furthermore, it lays a foundation for advancing nursing research and education, and provides valuable insights for policymakers to enhance nursing practice.

## 1 Background

In 2012, noncommunicable diseases were responsible for approximately 38 million out of the 56 million reported deaths worldwide, of these, heart failure (HF) accounted for about half of the fatalities, highlighting its significant impact on global mortality [1]. Similar to the current situation in developed nations, HF is expected to surpass all other causes of death in most emerging countries by 2020 [2, 3]. Obesity, hypertension, and diabetes, which are prevalent risk factors, contribute significantly to the high proportion of heart failure cases in both developed and emerging countries [4]. Studies suggest that the severity of heart failure influences the frequency of readmissions, with more severe cases leading to higher hospitalization rates [5]. HF is a leading cause of hospital admissions and readmissions, posing a significant risk of mortality and increasing the likelihood of unexpected readmissions [1]. Over 20% of patients with HF thought to be readmitted within 30 days [6]. The high readmission rates can have a major impact on HF patients' quality of life [1]. In low‐ and middle‐income countries, including Ethiopia, HF develops at an earlier stage and contributes to a higher percentage of hospital readmissions compared to high‐income countries [3, 7]. Therefore, this study aimed to determine the number of readmissions per HF patients and identify the potential risk factors.

## 2 Methods

### 2.1 Study Area and Period

The study included 663 heart failure patients from randomly selected medical wards at referral hospitals in the Amhara region, using an interviewer‐administered questionnaire and chart review from October 2022 to February 2023. The region has 81 hospitals, 858 clinics, and 3560 health posts [8, 9]. The referral hospitals included in this study were Felege‐Hiwot, Tibebe‐Ghion, Debre‐Markos, and Debre Tabor in the Amhara region.

### 2.2 Study Design

A cross‐sectional study design was employed to examine the number of readmissions and their determinants among patients with heart failure in referral hospitals in the Amhara region, Northwest Ethiopia, in 2023. Due to the nature of the data, characterized by an excess of zeros and over‐dispersion, zero‐inflated negative binomial models were utilized to appropriately account for these features in the count data.

### 2.3 Data Collection Procedure and Tools

The data were collected from 663 patients with heart failure receiving care at referral hospitals in the Amhara region, Northwest Ethiopia, between September 2022 and February 2023. Patients were selected using simple random sampling. Data collection involved a structured chart review to extract clinical information, including demographic data, comorbidities, and treatment history. Additionally, interviewer‐administered questionnaires were used to gather information regarding self‐care practices, medication adherence, and social support. The tools used for the data collection were pre‐tested for reliability and validity before the study. The data were collected using validated data collection tools which had six parts. personal and socio‐demographic data, clinical characteristics, participants' laboratory results, and Self‐care practices [10], Oslo Social Support Scale (OSSS‐3) [11] and MARS‐10 rating scale [12].

### 2.4 Statistical Analysis

Statistical analyses were conducted using STATA version 16. The descriptive statistics, including means, standard deviations, medians, and frequencies, were calculated for all variables. The prespecified analysis used the zero‐inflated poisson regression model to analyze the count data with excessive zeros, as this model was chosen based on the study design and data characteristics which incorporates a logistic regression component to predict the excess zeros and a Poisson regression component to model the count outcomes [13], specifically the number of readmissions among heart failure patients.

However, after conducting the analysis, it was found that the data exhibited over‐dispersion (variance = 2.192, mean = 0.89), which violated the assumptions of the Poisson distribution. Therefore, the analysis was adjusted to use a zero‐inflated negative binomial (ZINB) regression model as an exploratory analysis which is more appropriate for the over‐dispersed data [13], as it accommodates both excess zeros and over‐dispersion. The model integrates a logistic regression component for the excess zeros and a negative binomial regression component for the count outcomes. The model fit was evaluated using AIC and BIC [14]. The relationship between independent variables and the number of readmissions was assessed using Incidence Rate Ratios (IRR) with 95% confidence intervals, with statistical significance set at *p* < 0.05 (two‐sided). Additionally, exploratory subgroup analyses were conducted to examine variations by factors such as age, sex, body mass index, pulse rate, respiratory rate, social support, and medication history.

This study was conducted in accordance with ethical standards and approved by the *Medicine and Health Science College* under approval number *RCSTTD/408/01/17* Informed consent was obtained from all participants prior to their inclusion in the study. Participants were fully informed of the purpose of the study, their right to withdraw at any time without consequence, and the confidentiality of their data. The study adhered to the CONSORT guidelines for transparency, ensuring the methodology and results were clearly reported for replication and systematic review purposes.

## 3 Results

### 3.1 Socio‐Demographic and Clinical Characteristics of Study Participants

Among the 663 patients with heart failure, 371 (56.0%) were female, and 484 (73.0%) were married, with a mean age of 51 (± 18.41). Approximately 6.81% of them had pulmonary tuberculosis (Table 1). The minimum number of readmissions among patients with heart failure was zero, and the maximum was eight. Moreover, the mean systolic and diastolic BP were 112.46 mmHg and 68.93 mmHg, respectively (Table 2).

**Table 1:** Socio‐demographic and clinical factors of readmission and its determinants among patients with heart failure at referral hospitals in Amhara region, Northwest Ethiopia; 2023.

			Frequency	Percent
**Factors**	Sex	Female	371	56.0
		Male	292	44.0
	PTB	Yes	31	6.81
		No	424	93.2
	Marital status	Divorced	39	5.9
		Married	484	73.0
		Single	85	12.8
		Widowed	55	8.3
	Educational status	College and above	90	13.6
		No formal education	397	59.9
		Primary school	107	16.1
		Secondary school	69	10.4
	Family history of HF	No	568	85.7
		Yes	95	14.3
	Stage of HF	Stage 1	5	0.8
		Stage 2	12	1.8
		Stage 3	419	63.2
		Stage 4	227	34.2
	NYHA functional class	Class 2	8	1.2
		Class 3	339	51.1
		Class 4	316	47.7
	Social support	Poor	512	77.2
		Moderate	132	19.9
		Strong	19	2.9
	medication adherence	Poor	328	49.5
		Poor	335	50.5
	Self‐care practice	Poor	351	52.9
		Good	312	47.1
	Type of HF medication	BB	218	32.9
		Digitalis	284	42.8
		Diuretics	89	13.4
		ACE/ARB	72	10.9
	Framingham criteria	PE	244	36.8
		Cardiomegaly	207	31.2
		NVD	115	17.3
		PND	21	3.2
		Rales	56	8.4
		S3 gallop	20	3.0
		Total	663	100.0

**Table 2:** Covariates of number of readmission and its determinants among patients with heart failure at referral hospitals in Amhara region, Northwest Ethiopia; 2023.

		Minimum	Maximum	Mean	Std. Deviation
**Dependent variable**	Number of readmission	0	8	0.89	1.497
**Covariates**	Age	15	94	51.25	18.410
	Body mass index	16	33	21.88	3.529
	Systolic BP	70	260	112.72	22.697
	Diastolic BP	24	150	69.14	13.067
	Respiratory rate	10	80	24.87	4.806
	Pulse rate	24	155	96.93	19.854
	Hemoglobin	2.50	36.00	13.4231	3.35706
	Serum sodium	13	214	137.00	12.768
	Serum potassium	0.56	136.00	4.3079	7.12482
	Serum creatinine	0.00	105.00	1.9190	8.06690

### 3.2 Number of Readmissions Per HF Patients

**Figure 1:**
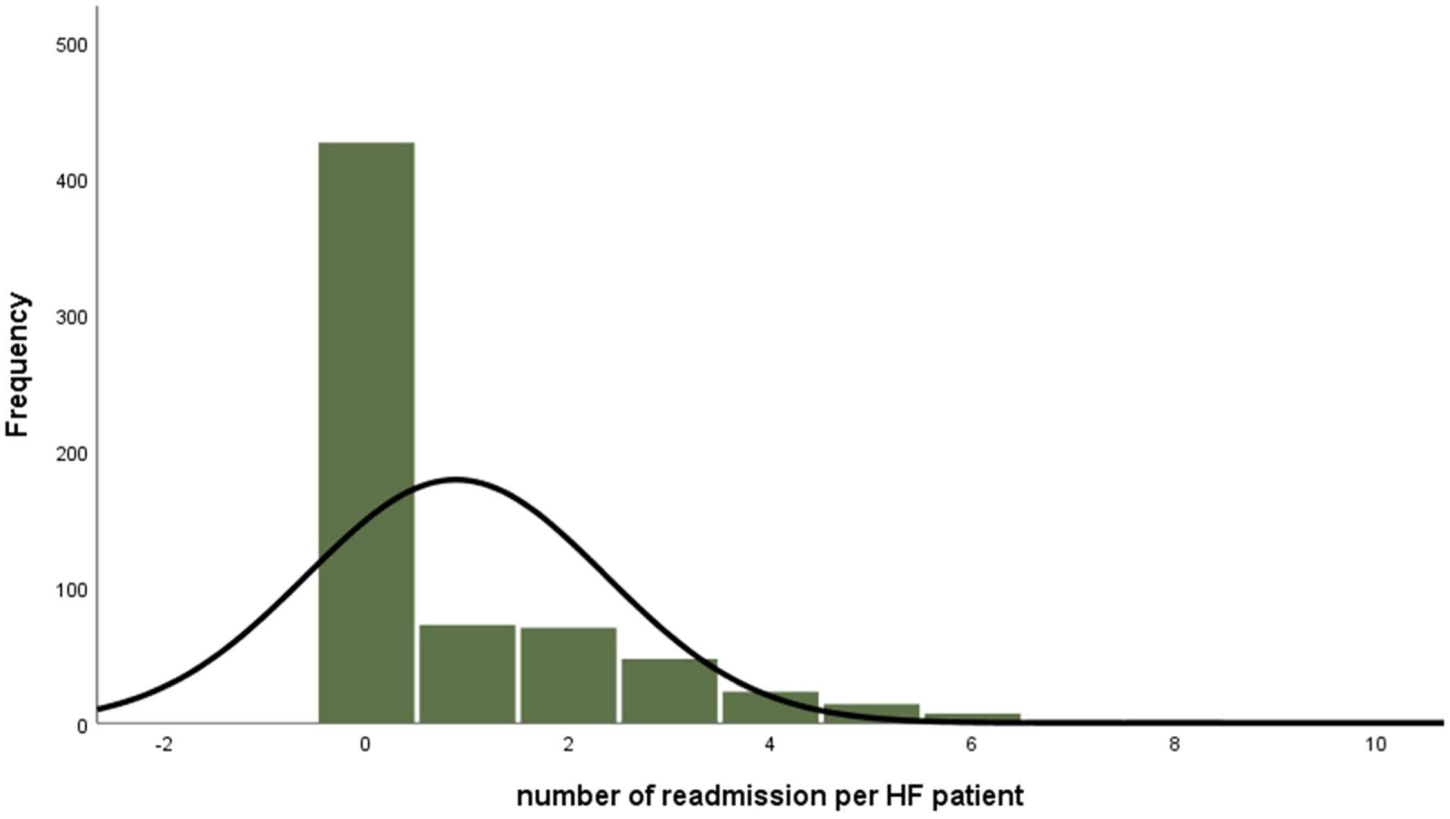
Histogram‐graph of number of readmissions per HF patients.

As can be seen, from Table 3 35.7% of patients with HF experience at least one readmission with a sample mean of 0.89 and variance of 2.19 (Table 3). The general pattern of readmissions per patient is heavily skewed to the right and has an excessive amount of zeros (Figure 1).

**Table 3:** Frequency and percentage distribution of number of readmission and its determinants among patients with heart failure at referral hospitals in Amhara region, Northwest Ethiopia; 2023.

Number of readmission per HF patient	Frequency	Percent
0	426	64.3
1	72	10.9
2	70	10.6
3	47	7.1
4	23	3.5
5	14	2.1
6	7	1.1
7	2	0.3
8	2	0.3
Total	663	100.0
Minimum	0	
Maximum	8	
Mean	89	
Variance	2.19	
Skewness	1.88	

As shown in Table 4, respiratory rate (RR) is significantly associated with the number of readmissions per patient. Specifically, the expected number of readmissions increased by 1.5% for every unit increase in RR (breaths per minute) (Coef. = 0.015; 95% CI: 0.00, 0.031; *p* = 0.044). Additionally, for every 1‐month increase in the duration of medication intake, the expected number of readmissions per patient decreased by 1.1% (Coef. = −0.01; 95% CI: −0.016, −0.0051; *p* < 0.0001), while holding all other variables in the model constant. Regarding the number of readmissions in the “always zero“ group of patients with heart failure who had poor social support had a 60% higher likelihood of not being in the ‘always‐zero’ group (Coef. = −1.595; 95% CI: −0.016, −0.005; *p* < 0.041). This indicates a significantly lower likelihood of readmission in the low social group due to access issues, highlighting a key health inequality.

**Table 4:** Parameter estimates of ZINB regression model among patients with heart failure at selected referral hospitals of Amhara region, 2023.

Count model coefficients (negbin with log link)						
Number of readmission	IRR.	Std. Err.	*z*	*P* > *z*	[95% Conf.	Interval]
**Number of readmission**						
**Sex**						
Female	−0.0270463	0.1407829	−0.19	0.848	−0.3029757	0.2488832
Male (ref)						
**Family history of HF**						
No	−0.0818296	0.156778	−0.52	0.602	−0.3891088	0.2254496
Yes (ref)						
**Education**						
College and above	0.5490596	0.2970542	1.85	0.065	−0.0331559	1.131275
No formal education	−0.0885132	0.2134577	−0.41	0.678	−0.5068826	0.3298562
Primary school	−0.4271887	0.2178605	−1.96	0.08	−0.8541875	−0.0001899
Secondary school (ref)						
**Stage of HF**						
Stage 2	0.0820675	0.7342817	0.11	0.911	−1.357098	1.521233
Stage 3	−0.1453738	0.481832	−0.3	0.763	−1.089747	0.7989995
Stage 4	−0.2448332	0.4853382	−0.5	0.614	−1.196079	0.7064122
Stage 1 (ref)						
**Social support**						
Poor	0.1706065	0.6632146	0.26	0.797	−1.12927	1.470483
Moderate	0.1019159	0.6722723	0.15	0.88	−1.215714	1.419545
Strong (ref)						
**Self‐ care practice**						
poor	0.0402067	0.1232299	0.33	0.744	−0.2013194	0.2817329
Good (ref)						
Body mass index	0.0358061	0.0213577	1.68	0.094	−0.0060542	0.0776665
Respiratory rate	0.0154837	0.0076926	2.01	**0.044**	0.0004064	0.030561
Pulse rate	−0.0024282	0.0028418	−0.85	0.393	−0.0079981	0.0031417
Duration of medication intake	0.0107112	0.0028843	3.71	**0.0001**	0.0163644	0.05058
Hemoglobin	−0.0212857	0.0169621	−1.25	0.21	−0.0545309	0.0119595
Serum potassium	0.0702924	0.0487471	1.44	0.149	−0.0252501	0.165835
Serum creatinine	−0.006113	0.0049025	−1.25	0.212	−0.0157217	0.0034957
_cons	−0.052737	1.042618	−0.05	0.96	−2.09623	1.990756
**inflate**						
**Sex**						
female	0.3563647	0.2868017	1.24	0.214	−0.2057563	0.9184858
Male (ref)						
**Family history of HF**						
No	0.4206392	0.3423762	1.23	0.219	−0.2504059	1.091684
Yes (ref)						
**Educational status**						
College and above	1.917893	0.6320176	3.03	0.002	0.6791614	3.156625
No formal education	0.3702433	0.501561	0.74	0.46	−0.6127981	1.353285
Primary school	0.0892595	0.5158982	0.17	0.863	−0.9218825	1.100401
Secondary school (ref)						
**Stage of HF**						
Stage 1 (ref)						
Stage 2	1.56772	1.794563	0.87	0.382	−1.94956	5.084999
Stage 3	1.056437	1.56955	0.67	0.501	−2.019825	4.132699
Stage 4	0.9871345	1.581299	0.62	0.532	−2.112154	4.086423
**Social support**						
Poor	−1.594966	0.9131699	−1.75	**0.041**	−3.384747	0.1948138
Moderate	−0.7841152	0.9324045	−0.84	0.4	−2.611595	1.043364
Strong (ref)						
**Self‐care practice**						
Poor	−0.4092432	0.2387236	−1.71	0.086	−0.8771329	0.0586465
Good (ref)						
Body mass index	0.1153165	0.0414054	2.79	**0.004**	0.0341634	0.1964696
Respiratory rate	0.0036628	0.0224326	0.16	0.87	−0.0403044	−0.04763
Pulse rate	−0.0127085	‐0.0060548	−2.1	**0.036**	−0.0245757	−0.008412
Duration of medication intake	−0.0064694	0.006211	−1.04	0.298	−0.0186428	0.005704
Hemoglobin	0.0017757	0.0327868	0.05	0.957	−0.0624852	0.0660366
Serum potassium	0.1372274	0.1005604	1.36	0.172	−0.0598674	0.3343223
Serum creatinine	−0.0460511	0.0358494	−1.28	0.199	−0.1163145	0.0242124
_cons	−2.708443	2.264821	−1.2	0.232	−7.147411	1.730524
/lnalpha	−14.78995	1664.181	−0.01	0.993	−3276.525	3246.945
alpha	3.77E‐07	0.0006281			0	.

*Note:* Vuong test of ZINB versus standard negative binomial: *z* = 6.41 Pr. > z = 0.0000.

On the other hand, the odds of being in the always zero group decreased by 11.5% while their body mass index increased by 1 Kg/m^2^ (Coef. = 0.115: 95% CI: 0.034, 0.196),” *p* < 0.004”. Moreover, the odds of being in the always zero group decreased by 1.27% while their pulse rate increased with 1 beat per minute for Coef. = −0.013: 95% CI: −0.025, −0.001), “*p* < 0.036” (Table 4).

## 4 Discussion

In this study, 35.7% of patients with heart failure readmitted at list once, which is higher than the study conducted in Iran, Denmark & Iceland (25%) [15], Italy (25.1%) [16] and Ethiopia (26.40%) [17]. However, this finding is lower than the study conducted in Japan (28%) [18], Syria (28%) and China (39.5%) [1]. This may be related to differences in study design, socio‐demographic characteristics, setting, utilization of private hospitals, and awareness of self‐care practices. According to the findings of this study, respiratory rate is an important determinant of the number of readmissions among patients with heart failure. Specifically, the number of readmissions increased by 1.5% for every one‐unit increase in respiratory rate (breaths per minute) (Coef. = 0.016; 95% CI: 0.0004, 0.031; *p* < 0.044). It is widely known that increasing in respiratory rate induced by HF are a symptom of a deteriorating prognosis caused for unplanned readmission [19]. This finding is consistent with the study in USA [20], and Australia [21]. Furthermore, while their pulse rate increased by one unit of beats per minute, the odds of being in the always zero group decreased by 1.27% (Coef. = −0.013: 95% CI: −0.025, −0.008), “*p* < 0.036” while other variables in the model are constant. This finding is in line with other finding in USA [20], New York [22], UK [23], China [24]. On the other hand, the odds of being in the “always zero” group decreased by 11.5% for each unit increase in BMI (Kg/m²) (Coef. = −0.115; 95% CI: −0.0342, −0.196; *p* = 0.004), Japan [25, 26] and US [27]. Despite patients from low socio‐economic backgrounds being likely to have low adherence [28], this research demonstrated persistence in the trend of not being admitted due to access issues and growing health inequalities. However this finding is contradicted with the stud in China [24, 29] and Australia [21]. In addition, the number of readmissions in the “always zero” group among patients with heart failure and poor social support decreased by 59.4% compared to patients with heart failure and good social support (Coef. = −1.595; 95% CI: −0.016, −0.005; *p* = 0.041), controlling for other variables in the model. This may be related to the fact that patients with poor social support have lower treatment compliance but are less likely to be readmitted due to the unavailability of support groups to visit healthcare facilities, regardless of their illness which is contradicted with the study conducted in Netherland [30], Iran, Denmark and Iceland [15], Switzerland [31], and US [32] and China [1]. Additionally, for every 1‐month increase in the duration of medication intake, the number of readmissions increased by 1.1% (Coef. = 0.011; 95% CI: 0.016, 0.05; *p* < 0.0001), while holding all other variables in the model constant. This may be related to the long‐term nature of the illness, the number of medications the patient must take, their lack of compliance, and the potential for complications to arise over time. Of the 237 heart failure patients with at least one readmission, 27 died during the follow‐up, yielding a mortality rate of 11.39%. This highlights the substantial risk of mortality in this group and the importance of targeted interventions.

## 5 Conclusions

This study found significant readmission rates among HF patients. Factors such as respiratory rate and medication duration were linked to increased readmissions, while poor social support was associated with fewer readmissions. The high mortality rate underscores the need for targeted interventions to improve patient outcomes.

## Author Contributions

All authors made significant contributions to this study. M.G. and B.T. conceptualized the study, while the methodology was developed by M.G., H.A., and T.W. Software for data analysis was managed by H.A. and G.G., and validation was conducted by W.G., T.Y.A., and L.Y. M.G. and G.G. performed formal analysis, and the investigation was carried out by T.W and B.T. Resources were provided by G.D. and B.T., and data curation was handled by H.A. and W.G. The original draft of the manuscript was prepared by M.G. and B.T., with critical review and editing by T.W, G.G., and W. G. Visualization was developed by H.A. and L.Y., and supervision was provided by T.Y.A. and G.D. Project administration was managed by M.G., and funding acquisition was handled by G.D. M.G. served as the corresponding author.

## Ethics Statement

This study was conducted in accordance with ethical standards and approved by the Debre Markos University, *Medicine and Health Science College Institutional Research Ethics Review Committee*, under approval number *RCSTTD/408/01/17* Informed consent was obtained from all participants prior to their inclusion in the study. Participants were fully informed of the purpose of the study, their right to withdraw at any time without consequence, and the confidentiality of their data. The study adhered to the CONSORT guidelines for transparency, ensuring the methodology and results were clearly reported for replication and systematic review purposes.

## Conflicts of Interest

The authors declare no conflicts of interest.

## Transparency Statement

The lead author Mihretie Gedfew affirms that this manuscript is an honest, accurate, and transparent account of the study being reported; that no important aspects of the study have been omitted; and that any discrepancies from the study as planned (and, if relevant, registered) have been explained.

## Data Availability

All relevant data have been presented within the manuscript. The dataset supporting the conclusions of this article is available from the authors upon request.

## References

[1] T.‐K. Lin , B. C. Hsu , Y. D. Li , et al., “The Impact of Sources of Perceived Social Support on Readmissions in Patients With Heart Failure,” Journal of Psychosomatic Research 154 (2022): 110723. 35078080 10.1016/j.jpsychores.2022.110723

[2] D. S. Celermajer , C. K. Chow , E. Marijon , N. M. Anstey , and K. S. Woo , “Cardiovascular Disease in the Developing World,” Journal of the American College of Cardiology 60, no. 14 (2012): 1207–1216. 22858388 10.1016/j.jacc.2012.03.074

[3] H. R. Wurie and F. P. Cappuccio , Cardiovascular Disease in Low‐ and Middle‐Income Countries: An Urgent Priority (Taylor & Francis, 2012), 543–550. 10.1080/13557858.2012.778642 PMC7613448 23534502

[4] T. A. Gaziano , A. Bitton , S. Anand , S. Abrahams‐Gessel , and A. Murphy , “Growing Epidemic of Coronary Heart Disease in Low‐ and Middle‐Income Countries,” Current Problems in Cardiology 35, no. 2 (2010): 72–115. 20109979 10.1016/j.cpcardiol.2009.10.002 PMC2864143

[5] S. E. Epstein , J. Zhu , A. H. Najafi , and M. S. Burnett , “Insights Into the Role of Infection in Atherogenesis and in Plaque Rupture,” Circulation 119, no. 24 (2009): 3133–3141. 19546396 10.1161/CIRCULATIONAHA.109.849455

[6] P. Klainin‐Yobas , S. H. Ng , P. D. M. Stephen , and Y. Lau , “Efficacy of Psychosocial Interventions on Psychological Outcomes Among People With Cardiovascular Diseases: A Systematic Review and Meta‐Analysis,” Patient Education and Counseling 99, no. 4 (2016): 512–521. 27045976 10.1016/j.pec.2015.10.020

[7] C. Padilla , O. Lobos , E. Hubert , et al., “Periodontal Pathogens in Atheromatous Plaques Isolated From Patients With Chronic Periodontitis,” Journal of Periodontal Research 41, no. 4 (2006): 350–353. 16827731 10.1111/j.1600-0765.2006.00882.x

[8] E. G. Mekonen , M. H. Gebrie , and S. M. Jemberie , “Magnitude and Associated Factors of Medication Administration Error Among Nurses Working in Amhara Region Referral Hospitals, Northwest Ethiopia,” Journal of Drug Assessment 9, no. 1 (2020): 151–158. 33235815 10.1080/21556660.2020.1841495 PMC7671667

[9] A. Getie , A. Wondmieneh , and T. Gebremeskel , Implementation of Nursing Process Among Nurses at Woldia Referral Hospital, Northern Ethiopia: An Institution‐Based Cross‐Sectional Study. 2021.

[10] A. Baymot , D. Gela , and T. Bedada , “Adherence to Self‐Care Recommendations and Associated Factors Among Adult Heart Failure Patients in Public Hospitals, Addis Ababa, Ethiopia, 2021: Cross‐Sectional Study,” BMC Cardiovascular Disorders 22, no. 1 (2022): 275. 35715744 10.1186/s12872-022-02717-3 PMC9206252

[11] R.‐D. Kocalevent , L. Berg , M. E. Beutel , et al., “Social Support in the General Population: Standardization of the Oslo Social Support Scale (OSSS‐3),” BMC Psychology 6, no. 1 (2018): 31. 30016997 10.1186/s40359-018-0249-9 PMC6050647

[12] N. O. Adomako , A. F. A. Marfo , M. N. A. Opare‐Addo , N. Nyamekye , and F. T. Owusu‐Daaku , “Blood Pressure Control, Accessibility, and Adherence to Antihypertensive Medications: Patients Seeking Care in Two Hospitals in the Ashanti Region of Ghana,” International Journal of Hypertension 2021 (2021): 1–9. 10.1155/2021/9637760 PMC8302388 34327016

[13] C. X. Feng and L. Li , “Modeling Zero Inflation and Overdispersion in the Length of Hospital Stay for Patients With Ischaemic Heart Disease,” Advanced Statistical Methods in Data Science (2016): 35–53.

[14] K. H. Lee , C. Pedroza , E. B. C. Avritscher , R. A. Mosquera , and J. E. Tyson , “Evaluation of Negative Binomial and Zero‐Inflated Negative Binomial Models for the Analysis of Zero‐Inflated Count Data: Application to the Telemedicine for Children With Medical Complexity Trial,” Trials 24, no. 1 (2023): 613. 37752579 10.1186/s13063-023-07648-8 PMC10523642

[15] M. Shamali , B. Østergaard , E. K. Svavarsdóttir , M. Shahriari , and H. Konradsen , “The Relationship of Family Functioning and Family Health With Hospital Readmission in Patients With Heart Failure: Insights From an International Cross‐Sectional Study,” European Journal of Cardiovascular Nursing 22, no. 3 (2023): 264–272. 35881489 10.1093/eurjcn/zvac065

[16] M. Canepa , L. Leporatti , L. Persico , et al., “Frequency, Characteristics and Prognostic Impact of Hospital Readmissions in Elderly Patients With Heart Failure: A Population Study From 2013 to 2017 in Liguria, Northern Italy,” International Journal of Cardiology 363 (2022): 111–118. 35728700 10.1016/j.ijcard.2022.06.052

[17] B. A. Anleye , P. K. Kumar , and A. H. Endris , “Unplanned Readmission to Hospital and Its Predictors in Heart Failure Patients, Ethiopia: A Retrospective Cohort Study,” medRxiv (2022): 2022.

[18] Y. Sun , M. Iwagami , J. Komiyama , et al., “The Effect of Home Care Support Clinics on Hospital Readmission in Heart Failure Patients in Japan,” Journal of General Internal Medicine 38 (2023): 2156–2163. 36650335 10.1007/s11606-023-08030-9 PMC10361922

[19] P. Barthel , R. Wensel , A. Bauer , et al., “Respiratory Rate Predicts Outcome After Acute Myocardial Infarction: A Prospective Cohort Study,” European Heart Journal 34, no. 22 (2013): 1644–1650. 23242188 10.1093/eurheartj/ehs420

[20] M. K. Bennett , M. Shao , and E. Z. Gorodeski , “Home Monitoring of Heart Failure Patients at Risk for Hospital Readmission Using a Novel Under‐The‐Mattress Piezoelectric Sensor: A Preliminary Single Centre Experience,” Journal of Telemedicine and Telecare 23, no. 1 (2017): 60–67. 26670209 10.1177/1357633X15618810 PMC5221726

[21] J. Howie‐Esquivel , K. Dracup , M. A. Whooley , et al., “Rapid 5 Lb Weight Gain Is Not Associated With Readmission in Patients With Heart Failure,” ESC Heart Failure 6, no. 1 (2019): 131–137. 30353706 10.1002/ehf2.12370 PMC6351885

[22] M. V. Habal , P. P. Liu , P. C. Austin , et al., “Association of Heart Rate at Hospital Discharge With Mortality and Hospitalizations in Patients With Heart Failure,” Circulation. Heart Failure 7, no. 1 (2014): 12–20. 24297690 10.1161/CIRCHEARTFAILURE.113.000429

[23] A. Vazir , B. Claggett , P. Jhund , et al., “Prognostic Importance of Temporal Changes in Resting Heart Rate in Heart Failure Patients: An Analysis of the CHARM Program,” European Heart Journal 36, no. 11 (2015): 669–675. 25368202 10.1093/eurheartj/ehu401

[24] H. Saito , M. Shoji , I. Taki , et al., “Five Prognostic Factors for Readmission in Patients Over 75 Years Old With Worsening Heart Failure,” The Showa University Journal of Medical Sciences 32, no. 1 (2020): 33–42.

[25] T. Umehara , A. Kaneguchi , N. Katayama , et al., “Frailty in Elderly Patients With Acute Heart Failure Increases Readmission,” Heart & Lung: The Journal of Critical Care 57 (2023): 102–109. 36126425 10.1016/j.hrtlng.2022.08.021

[26] M. Ito , H. Wada , K. Sakakura , et al., “Clinical Characteristics and Mid‐Term Outcomes of Non‐Elderly Obese Patients With Acute Decompensated Heart Failure in Japan,” International Heart Journal 59, no. 4 (2018): 766–771. 29794377 10.1536/ihj.17-410

[27] Z. L. Cox , P. Lai , C. M. Lewis , and J. Lindenfeld , “Body Mass Index and All‐Cause Readmissions Following Acute Heart Failure Hospitalization,” International Journal of Obesity 44, no. 6 (2020): 1227–1235. 31863028 10.1038/s41366-019-0518-6

[28] A. Mukhopadhyay , S. Blecker , X. Li , et al., “Neighborhood‐Level Socioeconomic Status and Prescription Fill Patterns Among Patients With Heart Failure,” JAMA Network Open 6, no. 12 (2023): e2347519. 38095897 10.1001/jamanetworkopen.2023.47519 PMC10722333

[29] M. Yang , L. Tao , H. An , et al., “A Novel Nomogram to Predict All‐Cause Readmission or Death Risk in Chinese Elderly Patients With Heart Failure,” ESC Heart Failure 7, no. 3 (2020): 1015–1024. 32319228 10.1002/ehf2.12703 PMC7261546

[30] C. Annema , M.‐L. Luttik , and T. Jaarsma , “Reasons for Readmission in Heart Failure: Perspectives of Patients, Caregivers, Cardiologists, and Heart Failure Nurses,” Heart & Lung: The Journal of Critical Care 38, no. 5 (2009): 427–434. 19755193 10.1016/j.hrtlng.2008.12.002

[31] F. Cilla , I. Sabione , and P. D'Amelio , “Risk Factors for Early Hospital Readmission in Geriatric Patients: A Systematic Review,” International Journal of Environmental Research and Public Health 20, no. 3 (2023): 1674. 36767038 10.3390/ijerph20031674 PMC9914102

[32] M. R. Sterling , J. B. Ringel , L. C. Pinheiro , et al., “Social Determinants of Health and 30‐day Readmissions Among Adults Hospitalized for Heart Failure in the REGARDS Study,” Circulation. Heart Failure 15, no. 1 (2022): 008409. 10.1161/CIRCHEARTFAILURE.121.008409 PMC8849604 34865525

